# Non-Contact Shear Wave Generation and Detection Using High Frequency Air-Coupled Focused Transducer and Fiber Optic Based Sagnac Interferometer for Mechanical Characterization

**DOI:** 10.3390/s22155824

**Published:** 2022-08-04

**Authors:** Gui Chen, Jinjun Xia

**Affiliations:** Department of Electrical & Computer Engineering, Lawrence Technological University, Southfield, MI 48075, USA

**Keywords:** non-contact characterization, non-contact modulus measurement, air-coupled transducer, shear wave generation, shear wave detection, sagnac system, air-coupled transducer calibration, PZT transducer, mechanical characterization

## Abstract

In shear wave-based material mechanical characterization, the transmit/receiver transducer is generally in contact with the material through a coupling medium. In many applications, especially in biological tissue-related characterization, the application of the coupling medium and the contact method are not ideal, sometimes even unacceptable, due to contamination or stress response concerns. To avoid contact, we developed a 1 MHz air-coupled focused PZT transducer as a moderate pressure generator that could induce a shear wave in soft material and a fiber optic-based Sagnac system for the detection of the propagating shear wave. A calibration indicated that the fabricated air-coupled focused PZT transducer could generate pressure above 1 KPa within its focal range. This pressure is three to five times as much as the pressure generated by a 1 MHz air-coupled transducer currently available on the market. The integrated system was demonstrated through shear wave generation by the fabricated air-coupled PZT transducer and shear wave detection by the fiber optic Sagnac system in a nylon membrane. The results demonstrated the capability of the integrated system in non-contact material mechanical characterization, such as in material modulus measurement.

## 1. Introduction

In ultrasound-based material mechanical characterization, mechanical stimulation and the detection of material response are two essential parts. In this high-resolution characterization, the ultrasound transducer is used to stimulate a broadband mechanical wave in the material under the inspection [1,2,3]. A coupling medium is generally needed to improve pressure delivery and wave generation efficiency. However, in many applications, especially in a biological tissue-related mechanical characterization, such as in eye tissue inspection, the application of a coupling medium is not ideal due to the concern of the stress response to the coupling medium or contamination to the tissue region. A direct non-contact wave excitation would be more preferred. With the same reason, a non-contact detection of the generated wave is also being pursued. The purpose of this study is to develop a system that can non-contact generate and detect a wave that can be used for material mechanical characterization, such as modulus measurement.

Both laser and air-coupled ultrasound can be used to non-contact excite a shear wave to propagate in material [4,5,6,7,8,9,10]. Laser excitation is easy to operate and control, and the bandwidth of the excited shear wave can be easily controlled through laser spot size and pulse duration. However, the operation safety concern prevents the wide acceptance of its application, in particular, in eye-related tissue mechanical characterization. In addition, the availability of a laser with proper wavelength is sometimes an issue. The laser wavelength must be matched with tissue optical absorption to generate a detectable shear wave. This can lead to a very expensive laser system requirement in certain tissue inspections. For example, in corneal application, an expensive UV laser system is needed because the corneal tissue absorption peak is in the UV region. These reasons make the applications with lasers as the wave excitation source very limited. On the contrary, an air-coupled ultrasound can generate a moderate pressure that is strong enough to excite a propagating shear wave in soft material. Its application is universal and appropriate for any soft tissues. Ultrasound is also more acceptable in the biomedical field. An air-coupled transducer for a shear wave excitation is chosen as our pursuit in this study.

An air-coupled transducer can be fabricated using PZT ceramic materials [11,12] or based on a capacitive micro-machined ultrasound transducer (CMUT) [13]. A CMUT transducer is more efficient in ultrasound emission in air due to its better acoustic impedance matching layer design and fabrication. However, its fabrication is more complicated and difficult in that it requires specialized equipment and skills. In addition, the generated pressure by a CMUT transducer is generally low. By comparison, a PZT based air-coupled transducer is easy to make, even in a general lab setting by a person with only basic training. In particular, due to the high electro-mechanical conversion efficiency, a PZT-based transducer can generate much higher pressure than a CMUT-based transducer. The major hindrance to making a good air-coupled PZT transducer is the realization of acoustic impedance matching due to the huge acoustic impedance difference between the PZT ceramic material and air. An acoustic impedance matching layer is required to make an efficient PZT transducer. Another consideration in the use of an air-coupled transducer is the large ultrasound attenuation in air. Ultrasound attenuation in air depends on wave frequency: the higher the frequency, the more attenuation there is. This is the reason that currently market-available air-coupled ultrasound transducers or research-based air-coupled transducers are limited to below the 0.5 MHz frequency range [14,15,16]. However, such a low frequency range limits the broadband wave excitation. In this study, we developed a 1 MHz air-coupled PZT transducer for a broadband shear wave excitation. The excitation beam size is also an important factor that determines the bandwidth of the generated shear wave. A smaller beam size can excite a broader bandwidth shear wave. Therefore, we chose a focused ultrasound for our development. The PZT transducer that we developed in this study can generate a line stripe-focused ultrasound field.

On the detection side of a non-contact mechanical characterization system, optical detection is a good choice. Optical coherence tomography (OCT)-based elastography (OCE) has been very successful in detecting shear waves for mechanical characterization, especially in the applications of biological tissue mechanical characterization [17,18,19]. However, an OCT system for an acoustic detection is very complicated. It requires difficult algorithm development and expensive components such as a high-speed camera, which limits its development in a general lab with limited resources. The complicated algorithms also increase the chance of artifacts in the processed detection results. To avoid the OCT drawbacks, we chose a fiber optic-based Sagnac detection system to detect shear waves. As an advantage, the Sagnac system directly detects an acoustic signal. It does not need to develop algorithms to convert the detected signal to an acoustic signal, which reduces the system complexity and potential artifacts induced by the software processing. The components required to construct a fiber optic Sagnac system are less expensive and can be afforded by many resource-limited labs. In particular, a fiber optic-based Sagnac system is very compact and portable and can be easily deployed to field applications.

This study is the first to integrate a high-frequency air-coupled focused PZT transducer with a compact fiber-optic-based Sagnac interferometer to achieve a broadband non-contact shear wave generation and detection. A full description of the fabrication and characterization of the home-made high frequency air-coupled focused PZT transducer was presented. The generated shear wave detection with a fiber optic Sagnac interferometer was demonstrated. The developed system demonstrated the potential in non-contact material mechanical characterization, such as modulus measurement.

## 2. Fabrication of Focused Air-Coupled PZT Transducer

**Figure 1:**
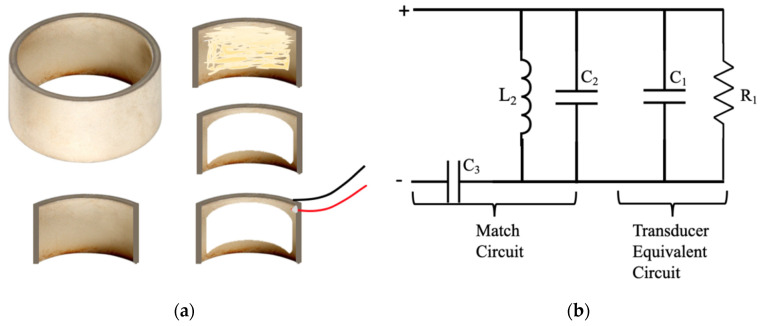
The focused air-coupled transducer: (**a**) Fabrication of the focused air-coupled transducer. (**b**) The equivalent circuit of the focused air-coupled transducer with the electrical impedance matching network.

A piezoelectric ceramic cylinder purchased from APC international, Ltd. (Catalog No. 42-1041, Mackeyville, PA, USA) was used to make the focused air-coupled transducer. The piezoelectric ceramic cylinder was made of a material similar to PZT-5A. The cylinder was made with high density and fine grain PZT ceramics that exhibited a high dielectric constant, high electromechanical coupling factor, high charge sensitivity and a high Curie point. The resonance frequency along the cylinder wall thickness dimension was 1 MHz. The frequency constant of the PZT cylinder was 2000 Hzm. The thickness of the cylinder was 2 mm. The inner radius of the cylinder was 13 mm, which defined the focal length of the fabricated transducer, i.e., 13 mm to the transducer inner surface. The cylinder was cut into a 90 degree curved arc structure with a height of one centimeter (see Figure 1a). Based on this structure, the finest focused ultrasound beam was 1 cm long and a 0.34 mm wide stripe under the diffraction limited condition.

To make the air-coupled transducer, acoustic impedance matching and electrical impedance matching are the two factors that need to be considered. To simplify the fabrication, we chose a ready membrane as the matching layer structure instead of a complicated designed microstructure used in a CMUT air-coupled transducer. Theoretically, the specific acoustic impedance of the matching layer should be the geometric mean of the acoustic impedance of the PZT material and air. The specific acoustic impedance of the PZT material we used is about 32.4 MRayl, while the specific acoustic impedance of air is about 416.5 Rayl. The specific acoustic impedance of the matching layer should be about 0.12 MRayl according to theory. This is a very low specific acoustic impedance that is even lower than the specific acoustic impedance of water, which is 1.5 MRayl. Based on available studies in the literature [11,12,20], a Nylon filter membrane was chosen for the matching layer. The specific acoustic impedance of the Nylon filter membrane (Cat No. 7404-004, Whatman Nylon Membrane Circles, Cytiva, Marlborough, MA, USA) we used is about 0.3 MRayl, which is close to a 0.12 MRayl value. The thickness of the matching layer is also an important factor that can affect wave transmission into air. The ideal thickness is a quarter wavelength of the transmitted ultrasound wave. For the nylon membrane we used, it has a thickness of about 150 µm, which is close to 210 µm: a quarter wavelength of the generated ultrasound wave in the membrane. For the electrical impedance matching, the transducer is modeled as a resistor parallel with a capacitor. An electrical matching network is designed to match the transducer network to have a 50 Ω impedance at the resonant frequency for the maximum power delivery from an RF power amplifier.

The fabrication step is shown in Figure 1a. After cutting from the PZT cylinder, the nylon matching layer was glued to the PZT transducer using an epoxy silicone (clear DAP, all-purpose adhesive sealant, 100% silicone) and placed in a clamping mold for 24 h for the curing. Two wires were soldered to the sides of the ultrasound transducer surface as the electrodes for applying the driving signal voltage. Figure 1b showed the electrical matching network we designed. At the resonant frequency, the impedance of the pure transducer without the electrical matching network was measured by a Vector Network Analyzer (Keysight Technologies, Colorado Springs, CO, USA). The measured complex impedance was equivalent to a parallel resistance and capacitance. The matching method was to use inductors and capacitors to tune out the reactance of the parallel capacitance, yielding a purely resistive load that was very nearly equal to the output impedance of the RF power amplifier. Here, we used a capacitor and an inductor connected to the transducer in parallel; then, another capacitor was used to achieve a total impedance of 50 ohm at the 1 MHz frequency to realize the electrical impedance matching. The measured 6 dB passband of the whole transducer was from 0.82 to 1.18 MHz with a bandwidth of 36% of the center frequency. The whole equivalent circuit of the transducer and the electrical impedance matching network is shown in Figure 1b.

## 3. Pressure Characterization of Focused Air-Coupled PZT Transducer

### 3.1. Calibration of Hydrophone Needle in Air Measurement

To calibrate the pressure generated by the focused air-coupled PZT transducer, a needle hydrophone (Precision Acoustics Ltd., Dorchester, Dorset, UK) was used to measure pressure. However, the needle hydrophone had been calibrated in water. In air, the coupling at the needle tip was different. To find the correcting coefficient for the measurement in air of the hydrophone, we had to calibrate the hydrophone measurement in air first.

**Figure 2:**
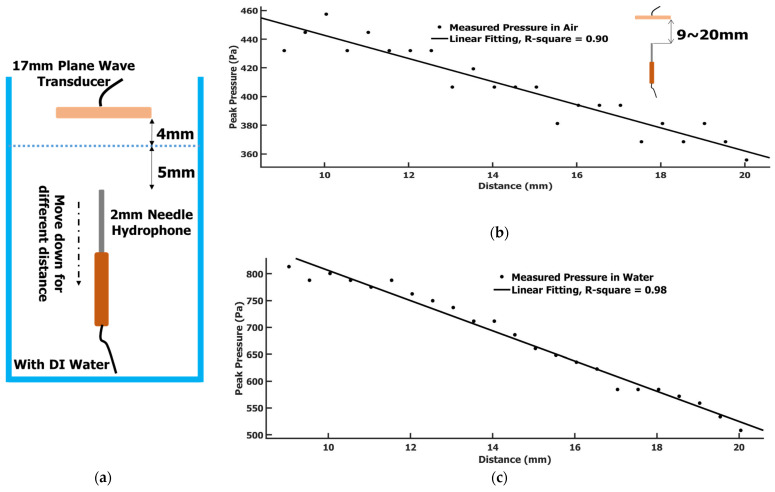
(**a**) The calibration setup; (**b**) The needle hydrophone in air measured pressure at different distance and its linear fitting (transmitter: Airmar air-coupled transducer, in air); (**c**) The needle hydrophone in water measured pressure at different distance and its linear fitting (transmitter: Airmar air-coupled transducer, in air).

The principle is based on the measurement of ultrasound plane wave attenuation in air and water and the plane wave reflection/transmission at the air/water interface with the help of a curve fitting. A plane wave was generated with a commercial air-coupled transducer (Airmar transducer) (1 MHz to 17 mm, AIRMAR Technology Company, Milford, NH, USA). Since it was an air-coupled transducer, it could be used as a wave transmitter/receiver without any coupling media. This Airmar transducer was a round flat shape with a 17 mm diameter. With such a shape and size, the wave sent out by this transducer into air could be safely treated as a plane wave. The Airmar transducer was only used as a wave transmitter in this experiment. It is worth mentioning that the Airmar transducer was the only air-coupled transducer that we could find in the market at such a high frequency (1 MHz). However, it cannot be used to generate a broadband shear wave. As a single transducer, the pressure it generated is not high enough, and the wave it generated is a plane wave. A function generator (AFG 3022B, Tektronix, Beaverton, OR, USA) was used to send out a 4-cycle 1 MHz sinusoidal wave with a Pk-Pk voltage of 800 mV as the excitation wave source. This signal was further amplified by an RF power amplifier with a 50 dB gain (240 L Power Amplifier, Electronics & Innovation, Ltd., Rochester, NY, USA) before it was delivered to the Airmar transducer. A 2 mm diameter hydrophone needle system calibrated by Precision Acoustics LTD (Dorchester, UK) was used to measure the ultrasound pressure. In this paper, peak pressure is used. The definition of peak pressure is (maximum pressure – minimum pressure)/2. The Airmar transducer and the hydrophone needle were installed as a Tx/Rx pair in a water tank environment with or without water (Figure 2a). The Airmar transducer was faced down and toward the needle hydrophone. The position of the Airmar transducer was fixed. The needle hydrophone was installed on a vertical stage that could be precisely moved up and down to control the distance to the Airmar transducer.

**Figure 3:**
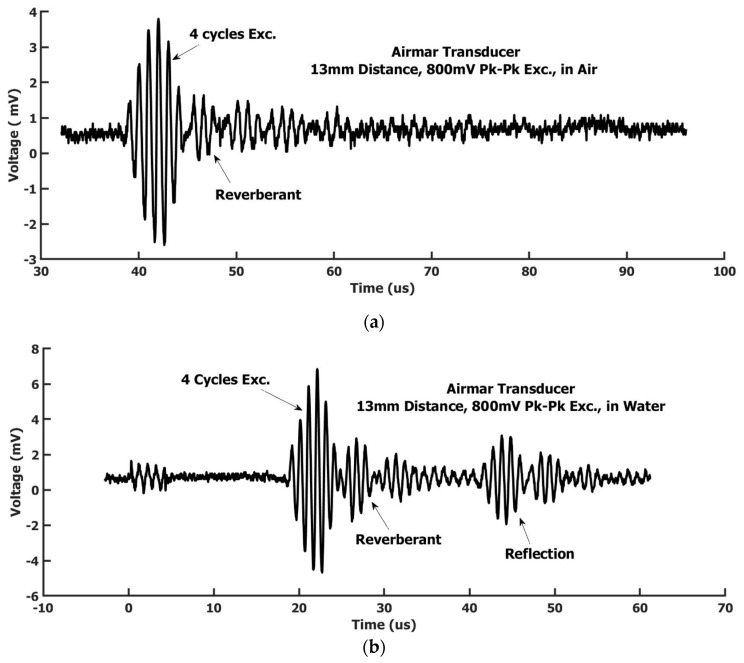
(**a**) The needle hydrophone in air-measured waveform (transmitter: Airmar air-coupled transducer, in air); (**b**) The needle hydrophone in water-measured waveform (transmitter: Airmar air-coupled transducer, in air).

*Step 1.* Without water in the tank, the needle hydrophone tip started at 9 mm below the Airmar transducer and could be vertically moved down to different positions. At each position, an oscilloscope was used to record the received ultrasound signal by the hydrophone needle. The step size between positions was 0.5 mm. Figure 3a showed a signal we recorded at 13 mm below the Airmar transducer. The signal basically displayed a 4-cycle signal with some ring-down and reverberant oscillation accompanied. The measured peak pressure data at different positions is shown in Figure 2b. The data appeared as a linear attenuation for this plane wave, not as an exponential attenuation. Because the distance was quite short, including the fitting extended range for the expectation value calculation, the total distance was about 15 mm. A linear approximation of this plane wave attenuation is certainly appropriate here. A linear regression was used for the curve fitting. A linear equation was obtained as:

$${Pressure}_{air}=-8.06*x_{distance}+523.3$$

From this equation, the peak pressure at 4 mm distance could be found as ${Pressure}_{air\_4mm}$ = 491.06 Pa. We also checked the measurement data by moving the hydrophone to 4 mm distance, and we measured a peak pressure of 508.26 Pa, which was about a 3% difference from the fitting data.

*Step 2.* Filled with de-ionized water in the water tank, the water surface to the Airmar transducer was 4 mm. The hydrophone needle was submerged in the water. The Airmar transducer was still kept in the same position as in step 1. The measurement positions of the needle hydrophone were also the same as in step 1, i.e., starting at 9 mm to the Airmar transducer and moving down by 0.5 mm each step. The 4 mm distance to the water surface of the Airmar transducer and the minimum distance of 5 mm to the water surface from the tip of the hydrophone needle (see Figure 2a) was to avoid the reflection wave from the water’s surface overlapping with the coming waves. Figure 3b showed a signal at a 13 mm distance. The correspondent signal magnitude measured in water was larger than in air. The ring-down effect and reverberant were similarly displayed as in the air. The peak pressure at different positions is shown in Figure 2c. The data distribution with distance displayed a better linear relationship than in air. The linear regression was obtained as:

$${Pressure}_{water}=-28.13*x_{distance}+1087$$

From this equation, the peak pressure at 4 mm distance can be found as ${Pressure}_{water\_4mm}$ = 974.48 Pa.

*Step 3.* To find the correcting coefficient for the measurement in air of the hydrophone, we could resort to the phenomenon of the reflection/transmission at the air–water interface. The plane wave was perpendicularly incident into water from air. We could use the transmission coefficient equation [21]:

$$\frac{{Pressure}_{water}}{{Pressure}_{air}}=T=\frac{2*Z_{water}}{Z_{water}+Z_{air}}=1.9994$$

where *Z*_water_ and *Z*_air_ were specific acoustic impedances of water and air, respectively, *Z*_water_ = 1.5 × 10^6^ Rayl and *Z*_air_ = 416.5 Rayl. Approximately, we could take the transmission coefficient value as 2. The air–water interface was 4 mm away from the Airmar transducer. Based on the pressure value in air obtained in step 1, the pressure incident into water at the same position would be Pressure_water_ = Pressure_air_ × *T* = 491.06 × 2 = 982.12 Pa. This was the theoretical value for the water environment we should obtain if the calibrated hydrophone was used in air to measure the pressure. However, according to step 2, the real measured pressure at the same position with the hydrophone in water was 974.48 Pa. The ratio between these two values would be the correcting coefficient for the measurement in air of the hydrophone. The correcting coefficient in air measurement of the hydrophone we obtained here was 974.48/982.12 = 0.99. Theoretically, all measured data in air should be multiplied by 0.99 to obtain the correct value. Using linear fitting 95% confidence bounds, the correcting coefficient was in the range of [0.9147, 1.0787]. Based on this result, we took the correcting coefficient as one for the hydrophone measurement in air. This result implies that the sensitivity of the hydrophone is the same for the measurement in air and in water. Therefore, the measurement error with hydrophone in air based on water environment calibration could be ignored. We treated the hydrophone in air measurement with the same accuracy as in the water measurement. We were confident to use this hydrophone needle to measure the transducer transmitter pressure in air.

### 3.2. Characterization of Focused Air-Coupled PZT Transducer

**Figure 4:**
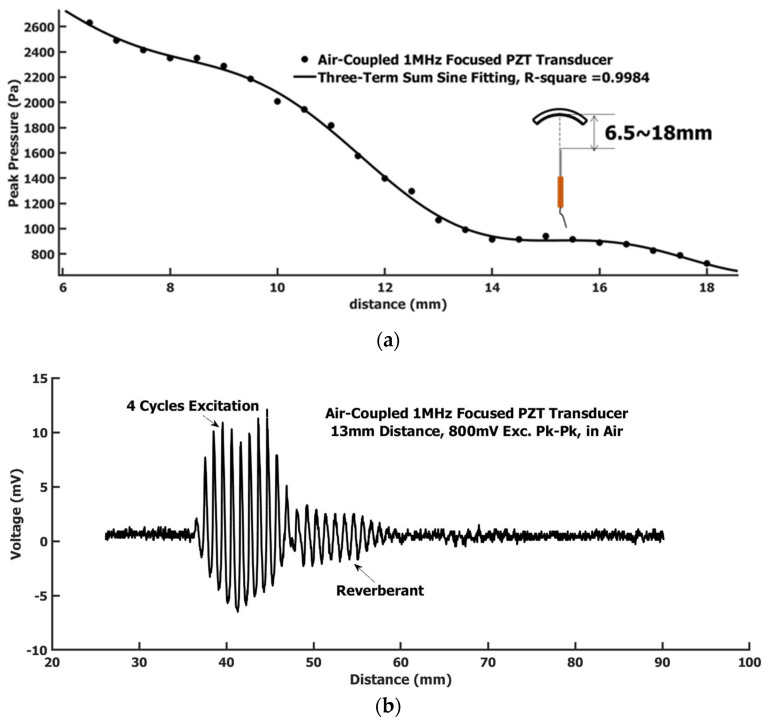
(**a**) The measured pressure at different distance by the needle hydrophone in air along the symmetrical center line of the focused air-coupled PZT transducer; (**b**) The needle hydrophone in air measured waveform at 13 mm distance generated by the focused air-coupled PZT transducer in air. The driving signal is a 4-cycle 1 MHz sine wave with Pk-Pk 800 mV with 50 dB gain before delivery to the transducer.

The field pressure along the symmetrical center line of the focused air-coupled PZT transducer was measured using the calibrated hydrophone needle. The setup sketch and results are shown in Figure 4a. The electrical wave excitation conditions were the same as used in the calibration procedure using the Airmar transducer for a comparison, i.e., 4-cycles 1 MHz sine wave with Pk-Pk voltage 800 mV and being amplified with a 50 dB gain with the same RF power amplifier. The pressures at different distances were measured from 6.5 to 18 mm with a 0.5 mm step size. Figure 4b was the waveform measured at a 13 mm distance. The waveform by the focused air-coupled PZT transducer showed more ring-down cycles. The reverberant also appeared. Ring-down and reverberant appeared more severe than in the Airmar transducer. Here, the PZT transducer was focused with an arc shape. The shown waveform was at the focal point position. The waves received by the needle hydrophone here, for except the waves from the center part of the transducer, were all obliquely incident to the hydrophone needle tip, which likely caused shear wave components in the detected signal. The wave field was much more complicated here than a simple plane wave in the Airmar case. Although the ring-down and reverberant signals were still at a high-frequency range of around 1 MHz, the outline of the ring-down and reverberant signals could be the low-frequency shear wave embedded. The peak magnitude of the signal was three times as high as that measured in the Airmar transducer. The pressure change with distance appeared as a three-term sine function summation trend as shown in Figure 4a. This is due to the transducer arc shape focus and the ultrasound attenuation in air. Overall, the ultrasound field intensity attenuated with the increase in distance. The field in the focal region of 13 mm away was smaller than the region closer to the transducer. This implied that the air attenuation was faster than the field focus. The pressure amplitude within the focal range was above KPa level.

**Figure 5:**
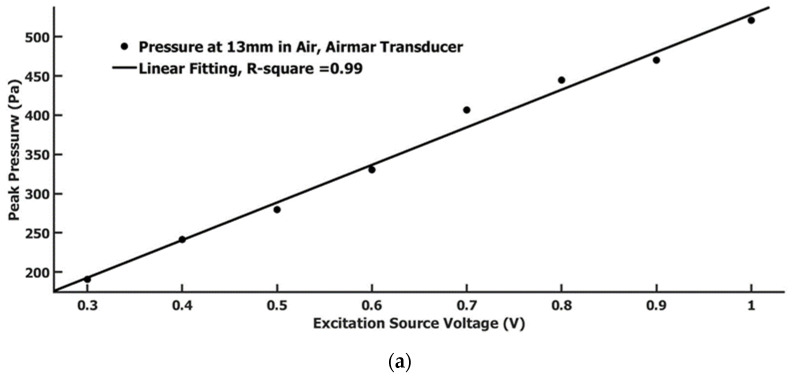

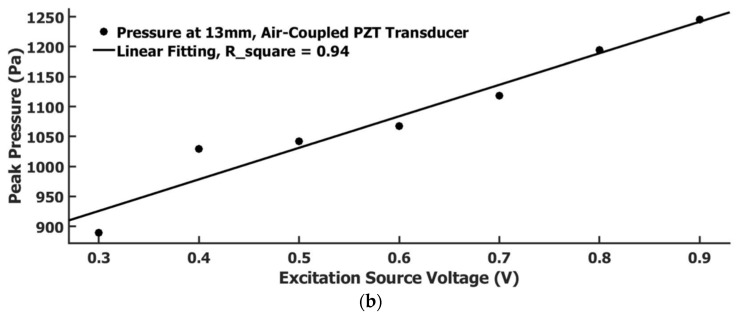
(**a**) The measured pressure under the different excitation peak-to-peak voltage for the Airmar air-coupled transducer; (**b**) The measured pressure under the different excitation peak-to-peak voltage for the focused air-coupled PZT transducer.

We also compared the generated pressure relationship with the excitation voltage for the Airmar transducer and focused air-coupled PZT transducer. The measurement position to the transducer is 13 mm for both transducers. The excitation voltage source was a 4-cycle 1 MHz sine wave with varied pk-pk voltage. The results are displayed in Figure 5a,b. Both showed a linear relationship. By comparison, the pressure generated by the focused air-coupled PZT transducer was three to four times as much as the Airmar transducer-generated pressure at a 13 mm distance.

## 4. Shear Wave Detection Using Fiber Optic Sagnac Interferometer

**Figure 6:**
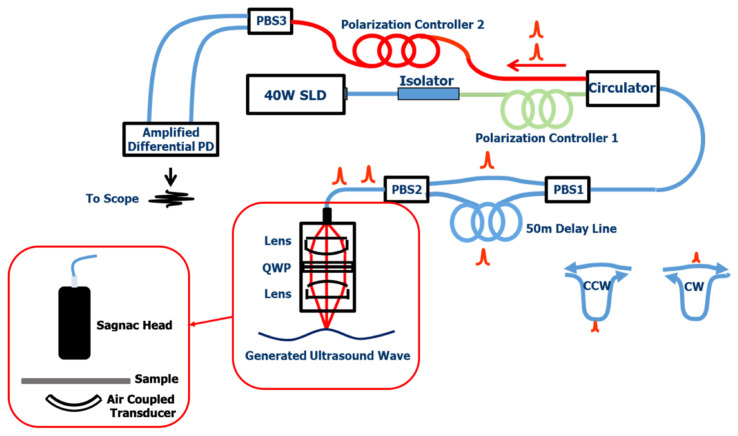
The Sagnac system principle diagram transducer.

A fiber optic Sagnac system was developed to detect the shear wave generated on a Nylon membrane. The membrane was a circular shape with a diameter of 47 mm and a thickness of about 150 micrometers. The sample was installed on a stage, and the focused air-coupled PZT transducer sent a longitudinal ultrasound wave onto the sample from the bottom side of the sample, as indicated in Figure 6. Figure 6 showed the fiber optic Sagnac system diagram. The detailed fiber optic Sagnac system principle and construction can be referred to [22,23]. Here, the purpose was to detect a shear wave, whose bandwidth was in the range of KHz level, so we limited our system detection bandwidth to about 2 MHz to reduce the system noise level. Therefore, the bandwidth delay line we used in this system was a 50 m long polarization maintained single mode fiber. The core concept in a Sagnac interferometer system is that the two interfering beams have the same optical path for a static surface; while for a vibration surface, the vibration information is encoded into the two interfering beams through a delay line, and the vibration speed is detected. The detected signal is directly an acoustic signal, not a displacement signal.

**Figure 7:**
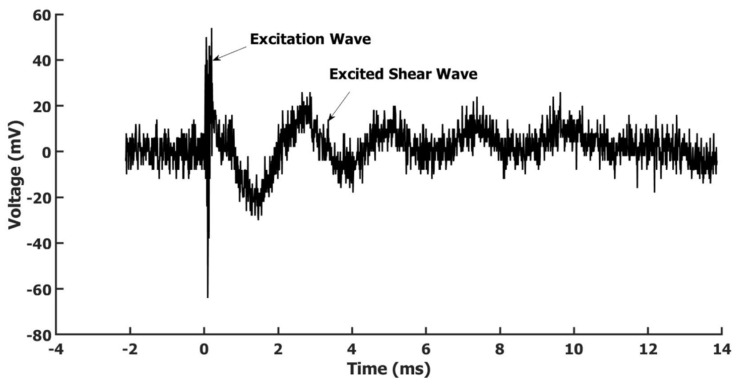
The excited shear wave on a nylon membrane by the air-coupled focused PZT transducer and measured by the fiber optic Sagnac system.

Figure 7 shows the result of the excited shear wave on the sample. The high-frequency excitation wave and the induced shear wave were both detected. The excitation wave was a sine wave with a linear frequency sweep from 0.825 to 1.275 MHz with a duration of 200 us. The product of duration and bandwidth was 90 to reduce the ring-down effect [24]. The excitation source Pk-Pk voltage was 800 mV; after a 50 dB power amplifier, about 253 V Pk-Pk voltage was delivered to the PZT transducer. To show the KHz level shear wave, the time scale was displayed as milliseconds, and the MHz excitation wave was compressed, as shown in the Figure 7. It can be seen, after the high-frequency longitudinal 200 us duration excitation wave, a shear wave was excited on the sample and propagated out from the source point. The excited shear wave mode is determined by the sample thickness, the width of the stripe-focused excitation ultrasound wave and the sound speed in the sample. The distance from the excitation PZT transducer to the sample surface determined the width of the focused ultrasound stripe. We obtained this result with a distance of about 10 mm. Compared to a system with a laser as an elastic wave excitation source and a phase-sensitive OCT-based receiving system [9], the system developed here has a better signal-to-noise ratio in the detected elastic wave. Of course, this could be due to the different measuring samples being used. The results demonstrated here indicated the potential of the integrated system in wave-based mechanical characterization. By measuring the phase speed of the generated shear wave, the modulus of the material can be measured according to the relationship of the shear wave phase speed and the material’s modulus [9].

## 5. Conclusions

A focused 1 MHz air-coupled PZT transducer for shear wave generation and a fiber optic Sagnac system for the generated shear wave detection have been developed. The integration of these two parts together realized non-contact wave generation and detection, which can be applied in material mechanical characterization, such as in modulus measurement. Based on the calibration, the constructed PZT transducer can generate KPa pressure within its focal range. This moderate pressure is enough to drive a vibration wave in soft material. As a demonstration, a shear wave induced by the developed focused air-coupled PZT transducer was detected by the developed fiber optic Sagnac system in a nylon membrane. The results demonstrated the capability in the material mechanical characterization with the developed integrated non-contact shear wave generation and detection system.

## Data Availability

Not applicable.
